# Cardiogenic shock due to pheochromocytoma-induced catecholamine crisis in a young woman

**DOI:** 10.1210/jcemcr/luag199

**Published:** 2026-07-27

**Authors:** Thirza van der Linden, Bjorn Dumont, Yalin Acardag, Johannes Hofland, Sjoerd Thijsse, Charlotte van Noord

**Affiliations:** Department of Internal Medicine, Maasstad Hospital, 3079 DZ Rotterdam, the Netherlands; Department of Internal Medicine, Maasstad Hospital, 3079 DZ Rotterdam, the Netherlands; Department of Cardiology, Maasstad Hospital, 3079 DZ Rotterdam, the Netherlands; Department of Internal Medicine, Section of Endocrinology, Erasmus Medical Center, 3015 GD Rotterdam, the Netherlands; Department of Intensive Care Medicine, Maasstad Hospital, 3079 DZ Rotterdam, the Netherlands; Department of Internal Medicine, Maasstad Hospital, 3079 DZ Rotterdam, the Netherlands

**Keywords:** pheochromocytoma, cardiogenic shock, catecholamine-induced cardiomyopathy, adrenal tumor, critical care

## Abstract

A pheochromocytoma is a rare catecholamine-secreting tumor that can cause life-threatening complications. Circulatory shock with a significant cardiogenic component is an uncommon initial presentation. This case describes a previously healthy 30-year-old woman presenting with vomiting, hyperglycemia, and shock. This rapidly progressed to requiring intubation, vasopressor therapy, and resuscitation following cardiac arrest. Imaging revealed a right adrenal mass and plasma metanephrines and normetanephrines were markedly elevated. After stabilization with α- and β-adrenergic blockade, the patient underwent successful adrenalectomy, confirming a pheochromocytoma-induced catecholamine crisis, with full recovery of cardiac function. This case illustrates the diversity of initial presentation of pheochromocytoma, the diagnostic challenges associated with measuring plasma (nor)metanephrines after resuscitation and the reversible nature of catecholamine-induced cardiomyopathy.

## Introduction

Pheochromocytoma is a rare catecholamine producing tumor of the adrenal gland, occurring in around 20 per million cases per year [1]. In 8% to 11% of these cases, the excess production of catecholamines leads to cardiomyopathy [2]. The presentation of pheochromocytoma can take many different forms, ranging from asymptomatic, to cardiogenic shock resulting in cardiac arrest [2, 3]. The low incidence and wide range of symptoms mean that the diagnosis pheochromocytoma is often not initially considered when patients present with cardiogenic shock. Prompt recognition, however, is essential due to the high mortality associated with delayed diagnosis [3, 4]. Cardiac function in these patients often improves with supportive care and tumor resection, demonstrating high recovery potential after adequate treatment [5]. This case report aims to present a patient with a pheochromocytoma induced cardiogenic shock, and to show that the clinical symptoms of pheochromocytoma can be misleading at first.

## Case presentation

A 30-year-old woman with no prior medical history presented to the emergency department (ED) with sudden onset of nausea, vomiting and abdominal pain. On arrival at the ED, vital signs were unstable showing marked hypotension (75/65 mm Hg) and tachycardia of 110 beats per minute with tachypnea of 35/min and hypothermia of 34.7°C.

## Diagnostic assessment

Initial physical examination revealed cold, mottled extremities and bilateral pulmonary rhonchi. Abdominal and neurological examinations were otherwise unremarkable. Laboratory testing (Table 1) showed severe metabolic acidosis with a pH of 6.97 (reference range, 7.32-7.38), lactate of 99 mg/dL (11 mmol/L) (reference range, 4.5-18 mg/dL [0.5-2.0 mmol/L]), and hyperglycemia of 594 mg/dL (33 mmol/L) (reference range, 74-110 mg/dL [4.1-6.1 mmol/L]), along with signs of renal failure. Pregnancy test was negative. Chest x-ray showed spotted bilateral consolidations across the lungs. Electrocardiogram showed a sinus tachycardia of 108 beats per minute, and ST depressions in leads II, III, augmented vector foot, V3 to V5, and minimal ST elevation in augmented vector left without Q waves. Before the administration of any vasopressors, the echocardiogram showed an almost akinetic left ventricle.

**Table 1:** Laboratory test results

Parameter	Result	Reference range
ALT	40 U/L	<34 U/L
GGT	25 U/L	<38 U/L
Albumin	36 g/L	35 to 52 g/L
Calcium	2.40 mmol/L (9.6 mg/dL)	2.20-2.65 mmol/L (8.8 to 10.6 mg/dL)
Corrected calcium	2.50 mmol/L (10 mg/dL)	2.20-2.65 mmol/L (8.8 to 10.6 mg/dL)
Chloride	96 mmol/L (340 mg/dL)	97-107 mmol/L (344 to 380 mg/dL)
Magnesium	1.36 mmol/L (3.3 mg/dL)	0.70-1.05 mmol/L (1.7 to 2.4 mg/dL)
Phosphate	3.20 mmol/L (9.9 mg/dL)	0.80-1.40 mmol/L (2.5 to 4.5 mg/dL)
Potassium	4.1 mmol/L (16 mg/dL)	3.5-5.0 mmol/L (13.7 to 19.5 mg/dL)
Sodium	136 mmol/L (313 mg/dL)	135-145 mmol/L (310 to 334 mg/dL)
Creatinine	135 μmol/L (1.53 mg/dL)	45-84 μmol/L (0.5 to 1.3 mg/dL)
eGFR (CKD-EPI)	45 mL/min/1.73 m^2^	>90 mL/min/1.73 m^2^
Leukocytes	15.7 × 10^9^/L	4.0-10.0 × 10^9^/L
CRP	2 mg/L	<10 mg/L
Glucose	33.9 mmol/L (610 mg/dL)	4.1 to 6.1 mmol/L (74 to 110 mg/dL)
HbA_1c_	39 mmol/mol (5.7%)	29 to 42 mmol/mol (4.8% to 6.0%)
Hb	8.3 mmol/L (13.4 g/dL)	7.5 to 10.0 mmol/L (12 to 16 g/dL)
Lactate	11 mmol/L (99 mg/dL)	0.5 to 2.0 mmol/L (4.5 to 18 mg/dL)
Base excess	−18 mmol/L	−3 to 3 mmol/L
Bicarbonate (HCO_3_^−^)	14 mmol/L (85 mg/dL)	23 to 27 mmol/L (140 to 165 mg/dL)
pH	6.97	7.32 to 7.38
pCO_2_	63 mm Hg (8.4 kPa)	42 to 50 mm Hg (4.7 to 6.0 kPa)
pO_2_	15 mm Hg (2.0 kPa)	75 to 100 mm Hg (10.0 to 13.3 kPa)

**Abbreviations:** ALT, alanine transaminase; GGT, γ-glutamyl transferase; eGFR (CKD-EPI), estimated glomerular filtration rate (Chronic Kidney Disease Epidemiology Collaboration); CRP, C-reactive protein; HbA_1c_, hemoglobin A_1c_; Hb, hemoglobin; pCO_2_, carbon monoxide partial pressure; pO_2_, oxygen partial pressure.

## Treatment

The patient initially received oxygen and fluids with vasopressors for stabilization. Intravenous (IV) insulin was started, and broad-spectrum antibiotics were given. The patient was transferred to the intensive care unit (ICU). She underwent aggressive resuscitation with IV fluids and required high doses of vasopressor support (arginine vasopressin, norepinephrine, epinephrine) and hydrocortisone. Despite maximal supportive measures and vasopressor therapy, she rapidly progressed to cardiac arrest. Advanced life support was initiated. Pulseless electrical activity was present from the onset and persisted throughout the cardiac arrest. During intubation, pink edema, highly suggestive of cardiogenic pulmonary edema, appeared in the oral cavity. After 15 minutes, return of spontaneous circulation (ROSC) was achieved.

After cardiac arrest a second echocardiogram was performed showing a left ventricular function ejection fraction (LVEF) of around 25%. This was under the additional administration of milrinone to improve LVEF. Following ROSC, the patient was treated in accordance with targeted temperature-management protocols. Anuria developed for which continuous venovenous hemodialysis was started. The patient was subsequently stabilized, allowing further diagnostic evaluation.

Contrast-enhanced computed tomography (CT) showed a 7.5-cm (2.95-inch) heterogeneous right adrenal mass (Fig. 1). Due to the large adrenal incidentaloma, the plasma metanephrines and normetanephrines were analyzed while patient was still receiving norepinephrine infusion in the ICU. Results showed 127-fold elevated normetanephrine and more than 242-fold elevated metanephrine levels (Table 2). However, these levels could have been falsely elevated due to stress and administered vasopressors.

**Figure 1:**
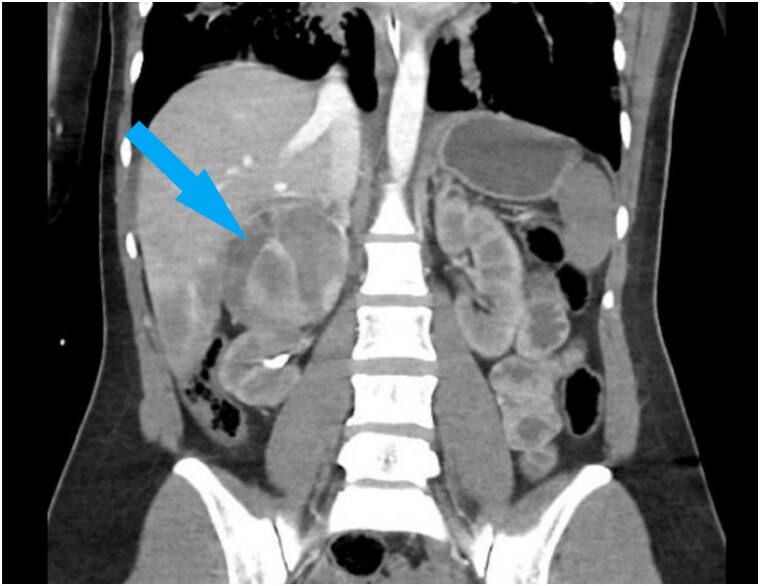
Computed tomography scan with coronal reconstruction in portal venous contrast phase shows a heterogeneously enhancing 7.5-cm (2.95-inch) right adrenal mass (arrow).

**Table 2:** Plasma metanephrines

Parameter	Result	Reference range
Day 1, after CPR		
Metanephrine	80.03 nmol/L (14 405 pg/mL)	0.07-0.33 nmol/L (13-59 pg/mL)
Normetanephrine	>136.5 nmol/L (>22 932 pg/mL)	0.23-1.07 nmol/L (39-180 pg/mL)
3-Methoxytyramine	15.29 nmol/L (2 553 pg/mL)	<0.17 nmol/L (<28 pg/mL)
Day 6		
Metanephrine	15.46 nmol/L (2 783 pg/mL)	
Normetanephrine	46.04 nmol/L (7 738 pg/mL)	
3-Methoxytyramine	1.77 nmol/L (296 pg/mL)	
1 wk after surgery		
Metanephrine	0.08 nmol/L (14 pg/mL)	
Normetanephrine	0.83 nmol/L (138 pg/mL)	
3-Methoxytyramine	<0.05 nmol/L (<8 pg/mL)	

**Abbreviation:** CRP, C-reactive protein.

The clinical picture of cardiogenic shock with a large adrenal mass in a young woman with no prior medical history led to the suspicion of pheochromocytoma-induced cardiogenic shock.

α-Adrenergic blockade with IV urapidil was initiated. The patient was transferred to a tertiary center. Six days after cardiac arrest plasma (nor)metanephrines were obtained again after vasopressors were stopped for more than 12 hours and urapidil was stopped for 2 hours. Metanephrine and normetanephrines improved but remained 47 and 43 times elevated, respectively (see Table 2). Oral α- and β-adrenergic blockers were initiated and titrated upward, but the patient continued to show daily fluctuations in blood pressure consistent with episodic catecholamine release. She subsequently underwent successful laparoscopic resection of the adrenal tumor.

Pathologic findings confirmed a 6-cm (2.36-inch) pheochromocytoma with intratumoral hemorrhage. The tumor was completely resected and plasma (nor)metanephrine levels normalized 1 week after surgery.

## Outcome and follow-up

Two weeks after presentation, the patient recovered with normalization of cardiac function; the LVEF was estimated to be 50%. After surgery the patient further recovered and underwent rehabilitation afterward. Three months after her first presentation, her echocardiogram showed fully recovered cardiac function with no abnormalities. Genetic testing revealed no abnormalities.

## Discussion

Pheochromocytoma is a rare but potentially life-threatening endocrine tumor that requires prompt recognition, appropriate diagnostic work-up, and dedicated treatment. Cardiogenic shock as the presenting manifestation of pheochromocytoma is rare but well documented in case reports [6, 7] and retrospective studies [5, 8].

Most common symptoms of pheochromocytoma are palpitations (58.1%), episodic headache (51.9%), and sweating (48.8%) [9].

The combination of these 3 symptoms has been described as the “classic triad” of pheochromocytoma. Despite being called the classic triad, with this specific combination of symptoms seen in up to 24% of patients, there is also a proportion of patients that are asymptomatic [10]. In our case the patient had no prior symptoms. Symptoms become more severe when a pheochromocytoma precipitates a catecholamine crisis, a rare endocrine emergency leading to life-threatening hemodynamic instability causing end-organ damage or dysfunction. In 39.5% of cases pulmonary edema occurred [11], in 47.9% of patients echocardiography revealed a low (<25%) LVEF [12], while hyperglycemia was observed in 25% to 75% [13].

Symptoms caused by supraphysiological levels of (nor)epinephrine are nonspecific to pheochromocytoma, highlighting the challenges associated with its timely diagnosis.

The 2023 European Society of Endocrinology clinical practice guidelines on the management of adrenal incidentalomas [12] recommend the measurement of plasma free (nor)metanephrines or urinary fractionated (nor)metanephrines as the preferred biochemical tests for the diagnosis of pheochromocytoma. This poses a diagnostic challenge in cases like ours, when vasopressors are administered surrounding resuscitation and the patient experiences stress resulting in high levels of sympathetic activity. These can lead to false-positive test results of plasma (nor)metanephrines.

The half-lives of metanephrine and normetanephrine are both less than 4 minutes [14]. Major guidelines do not specify an exact interval for testing, but practical recommendations are to delay testing until acute illness and recent catecholamine exposure have resolved [15-17]. However, this was not possible in our case. This makes the test of limited value in the acute setting.

When plasma metanephrines or urinary fractionated metanephrines are indicative of pheochromocytoma, CT is the first-choice imaging modality. A variety of medical treatments are possible, but the only definitive treatment is resection of the pheochromocytoma [12]. After tumor removal and with supportive care, cardiac function often improves [5, 18-20].

This case illustrates a rare first presentation of pheochromocytoma with cardiogenic shock due to excessive catecholamine production. This type of case may mimic diabetic ketoacidosis (DKA) or sepsis. Hyperglycemia, metabolic acidosis, and acute kidney injury are common but nonspecific features. In our case, the metabolic acidosis, hyperglycemia, absence of prior medical history, and young age led us to believe this was a case of DKA as a complication of a novel diabetes mellitus type 1. Only the incidental finding of a mass in the adrenal when performing a CT triggered the thought of a pheochromocytoma as an underlying cause of the clinical findings.

## Learning points

- In patients with unexplained cardiogenic shock, especially in the absence of prior cardiac disease, pheochromocytoma must be considered.
- Measurement of plasma free (nor)metanephrines for diagnosis of pheochromocytoma may be of limited value in the acute setting.
- Early diagnosis and multidisciplinary management are critical for survival. Imaging and biochemical confirmation followed by α-adrenergic blockage and timely surgical resection offer an excellent prognosis.

## Contributors

T.L., B.D., Y.A., J.H., S.T., and C.N. made substantial contributions to the conception or design of the work, drafting the work, or reviewing it critically for important intellectual content, final approval of the version to be published, and agreement to be accountable for all aspects of the work in ensuring that questions related to the accuracy or integrity of any part of the work are appropriately investigated and resolved. Y.A., C.N., S.T., and J.H. were involved in the diagnosis and management of the patient.

## Data Availability

Data sharing is not applicable to this article as no datasets were generated or analyzed during the current study.

## Abbreviations

CT: computed tomography
DKA: diabetic ketoacidosis
ED: emergency department
ICU: intensive care unit
IV: intravenous
LVEF: left ventricular function ejection fraction
ROSC: return of spontaneous circulation
